# Investigating the Dose-Response Relationship between Deep Breathing and Heart Rate Variability in Healthy Participants and Across-Days Reliability in Patients with Rheumatoid Arthritis and Systemic Lupus Erythematosus

**DOI:** 10.3390/s22186849

**Published:** 2022-09-10

**Authors:** Caroline Hundborg Liboriussen, Stine Søgaard Andersen, Sally Søgaard Andersen, Mette Kjeldsgaard Jensen, Mads Jochumsen, Salome Kristensen

**Affiliations:** 1 Department of Health Science and Technology, Aalborg University, 9220 Aalborg, Denmark; 2 Department of Rheumatology, Aalborg University Hospital, 9000 Aalborg, Denmark

**Keywords:** rheumatoid arthritis, systemic lupus erythematosus, deep breathing, heart rate variability, vagus nerve stimulation, neuromodulation, breathing, dose-response, reliability, inflammation

## Abstract

Rheumatoid Arthritis (RA) and Systemic Lupus Erythematosus (SLE) are associated with autonomic dysfunction, potentially through reduced vagus nerve tone. Vagus nerve stimulation has been proposed as an anti-inflammatory treatment, and it can be performed through deep breathing (DB) exercises. In this study, the dose-response relationship between DB exercises and heart rate variability (HRV) was investigated in healthy participants and reliability across days in patients with RA and SLE. On three separate days, 41 healthy participants performed DB for: 5, 15, or 30 min. On two separate days, 52 RA or SLE patients performed DB with the dose associated with the highest HRV increase in healthy participants. The HRV was estimated from ECG-recordings recorded prior and post the DB exercises. Increases in dose led to larger HRV-responses. Thirty minutes led to the largest HRV-response. In the RA and SLE patients, this dose increased the HRV-parameters consistently across the two days, indicating reliability. DB increases HRV in healthy participants and RA or SLE patients, which indicates stimulation of the vagus nerve. Of the tested durations, 30 min of DB was the optimal period of stimulation. A potential anti-inflammatory effect of DB exercises should be investigated in future studies.

## 1. Introduction

Rheumatoid Arthritis (RA) and Systemic Lupus Erythematosus (SLE) are chronic autoimmune diseases with systemic inflammation. RA affects 0.5–1.0% of adults in industrialised countries and the prevalence of SLE in adults ranges from 30 to 150 per 100,000, both diseases being more common in women [1,2]. Many therapeutic agents exist for treating RA and SLE. However, SLE-treatment still has high failure rates and toxicity, and SLE is associated with premature mortality [2]. Likewise, patients with RA have higher mortality compared to the general population [3], and despite different treatment options there are still patients with RA who continue to have signs and symptoms suggestive of inflammatory disease activity [4]. In addition, pain often remains problematic even in low levels of inflammation [5] and many patients experience side effects from the medicine, which can lead to withdrawal of the treatment [6]. These circumstances call for novel treatment strategies in the management of RA and SLE.

Studies have investigated the autonomic nervous system and found that patients with RA and SLE have an imbalance of their autonomic nervous system [7,8]. A systematic literature review by Adlan et al. found that 60% of patients with RA have an autonomic nervous system dysfunction—especially reduced parasympathetic activity and altered heart rate variability (HRV) [8]. HRV is a method used to evaluate cardiac autonomic nervous system function [8], especially vagus nerve tone [9]. A high HRV indicates good health and vitality [10], and reduced HRV, which is seen in patients with RA and SLE, is associated with increased mortality [7,8]. Autonomic imbalance precedes the development of RA in individuals at risk of developing arthritis [11], and seropositivity, disease activity, and pro-inflammatory cytokine levels are predictive of autonomic dysfunction [12]. Patients with more vagal activity generally respond better to anti-tumour necrosis factor therapy, and in healthy subjects decreased parasympathetic activity is also correlated with increased inflammatory status [6,13]. Studies also confirm the presence of autonomic dysfunction measured by HRV in patients with SLE [14,15].The link between the autonomic nervous system and the immune system has been investigated previously to explain the autonomic dysfunction. In this context, the vagus nerve has been investigated as the possible missing link [16].

The vagus nerve is part of the parasympathetic nervous system and controls visceral functions. However, recent data have suggested an anti-inflammatory role of the vagus nerve as well, which is believed to be mediated through three different pathways: (a) The hypothalamic-pituitary-adrenal axis, (b) the cholinergic anti-inflammatory pathway, and (c) the splenic sympathetic anti-inflammatory pathway [16]. The outcomes are inhibition of the pro-inflammatory cytokine tumor necrosis factor-α or increased cortisol, which results in an anti-inflammatory effect [16]. Knowing that patients with RA and SLE have an autonomic dysfunction, especially decreased parasympathetic function, an interesting perspective could be to investigate if stimulation of the vagus nerve could exert an anti-inflammatory effect and thereby improve disease activity in these patients.

One possible way of upregulating the vagus nerve tonus could be through vagus nerve stimulation (VNS). VNS is currently being used in the treatment of refractory epilepsy, migraine, cluster headache, and depression [17,18]. VNS can be both invasive and non-invasive. Invasive techniques involve implantable devices with a cuff electrode around the vagus nerve on the neck and non-invasive techniques include transcutaneous VNS (tVNS) of the auricular or the cervical branch of the vagus nerve. A study by Koopman et al. found that 21 days of invasive VNS through an implantable vagus nerve stimulator in 17 patients with RA inhibited tumor necrosis factor production and decreased the disease severity significantly [17]. However, when using invasive techniques such as surgical implantation of a VNS device there is always a risk associated with the general anaesthesia and a risk of postoperative complications [19]. Physiological methods of stimulating the vagus nerve also exist, such as deep breathing (DB), yoga, or meditation [20,21,22]. Therefore, if physiological methods were to be effective, they would often be preferred, due to fewer adverse events, no financial costs, and their transferability into clinical practice.

A way to modulate the vagus nerve without invasive procedures and equipment is through DB, where the vagus nerve is stimulated through the baroreflex: When blood pressure increases, the arterial baroreceptors are activated. This results in activation of the vagus nerve, which signals to the sinoatrial node in the heart, resulting in decreased heart rate. The same mechanisms are activated during DB, due to changes in the intrathoracic pressure, thereby affecting blood pressure and ultimately resulting in increased vagus nerve tone and increased HRV [23].

Several studies have investigated both yoga and breathing exercises in healthy participants and found an increase in HRV-parameters [24,25,26]. In a cross-over clinical trial with 25 healthy participants, Sharpe et al. found that different breathing interventions (2 × 10 min) increased the time domain HRV-parameters [24]. In 21 healthy participants, Tavares et al. found an increase in the time- and frequency domain HRV-parameters after guided breathing for 10 min [25]. In a cross-sectional study with 14 yoga practitioners and 14 non-yoga practitioners, Muralikrishnan et al. found that the time- and frequency domain HRV-parameters increased significantly more in yoga practitioners during one minute of controlled deep breathing [26]. Despite several studies having found increases in HRV-parameters after breathing exercises, others have found a lower parasympathetic nerve activity after controlled breathing in healthy subjects [27]. Sasaki et al. reported that five minutes of controlled breathing in 20 healthy subjects led to a reduced parasympathetic nerve activity evaluated through the frequency domain parameters [27]. To our knowledge, only one study investigated DB in patients with RA and SLE and found an increase in HRV [22]. Likewise, a study by Juel et al. found that the combination of deep slow breathing and tVNS could increase cardiac vagal tone in patients with chronic pancreatitis [20]. The field is therefore relatively unexplored, and no studies have examined for how long the DB exercises must be performed to modulate HRV in healthy participants and in patients with RA and SLE and the reliability of the method.

The aims of this study were to examine the dose-response of DB on HRV in healthy participants by investigating DB in 5, 15, and 30 min and to examine the reliability of the effect of DB on HRV in patients with RA and SLE. We hypothesize that the HRV increases when the duration of the DB is longer, and that a consistent increase in HRV is observed across days. Thus, the contribution of this paper is two-fold: (1) an investigation of the dose-response relationship of DB and the effect on HRV in healthy participants, and (2) the test-retest reliability of DB in patients with RA or SLE to investigate if similar changes are observed across days.

## 2. Materials and Methods

### 2.1. Participants

Forty healthy subjects were recruited for an interventional dose-response study, and 52 patients with RA or SLE were recruited for a study of across-days reliability. The healthy participants were recruited from the general community through social media. Patients with RA and SLE attending the Department of Rheumatology, Aalborg University Hospital, Denmark, were recruited during an outpatient visit at the department. For inclusion and exclusion criteria, see Table 1. Only healthy subjects were included in the first part of the study, and in the second part of the study patients with RA (based on the American College of Rheumatology (1987 or 2010) or European League Against Rheumatism (2010) classification criteria [28]) or SLE (according to American College of Rheumatology classification criteria for SLE [29] or Systemic Lupus International Collaborating Clinics Classification [30]) were included. The exclusion criteria included heart arrhythmias, to avoid incorrect HRV-data, and chronic lung disease, to make sure the subjects could complete the DB intervention. Demographic data about general health information, e.g., gender, age, and body mass index, were collected. Regarding patients with RA and SLE, information about disease activity, patient-reported outcome measures, and current treatment, was collected. Disease activity in patients with RA was based on the Disease Activity Score 28 CRP and Clinical Disease Activity Index while disease activity in patients with SLE was based on The Systemic Lupus Erythematosus Disease Activity Index. Patient-reported outcome measures included the Systemic Lupus Activity Questionnaire and the multidimensional health assessment questionnaire. Forty-one healthy participants were recruited, but one was excluded due to the development of severe psychiatric disease. Fifty-two patients with RA and SLE were recruited, but one was excluded due to several extrasystoles making the HRV-data incorrect. Table 2 outlines the characteristics about the participants.

### 2.2. Deep Breathing

The intervention was DB, consisting of four seconds of inspiration followed by six seconds of expiration, making the respiratory frequency six breaths per minute. This has previously been shown to increase HRV and the cardiac vagal tone in healthy subjects and patients with RA and SLE [20,22,31]. Prior to the intervention, the participants were instructed on how to perform the breathing exercises, and during the intervention the participants followed a visual cue to control the pace and to secure compliance and consistency among all participants. Healthy participants performed the intervention in 5, 15, and 30 min in a randomized order on 3 different days. The order of interventions was randomized to avoid a potential bias of a fixed order of the interventions. The randomization was generated using MATLAB’s “randperm” function (MathWorks^®^). Patients with RA and SLE performed the optimal dose of DB found in healthy participants on two different days to examine the reliability of the effect. The intervention was performed sitting, and each intervention was separated by a time interval of at least 24 h in both groups to avoid a potential carry-over effect.

### 2.3. Heart Rate Variability

HRV is a method used to evaluate cardiac autonomic nervous system function [8], especially vagus nerve tone [9], and it is defined as changes in the time intervals between consecutive heartbeats [9]. It was recorded twice before the intervention with a 5-min break between the measurements constituting the baseline and three times after the intervention (at 0–5, 12.5–17.5, and 25–30 min after the deep breathing intervention) to examine the interventional effect and the washout effect. A repeated-measures design was chosen such that potential changes due to the specific intervention were measured with respect to a baseline recorded on the same day. HRV was measured through an electrocardiogram (ECG), which was recorded in a bipolar derivation using electrodes (Ambu^®^ Whitesensor 0415M, Ambu A/S, Ballerup, Denmark) placed on the top part of the chest. The ECG was sampled with 250 Hz using a Cyton Bioamplifier (OpenBCI, New York City, NY, USA) and transmitted wirelessly to the computer for offline analysis. The ECG was recorded for 5 min in compliance with HRV guidelines [32].

### 2.4. Data Analysis

The recorded ECG was processed using a custom-made program (Mads Jochumsen, Aalborg University) where the data were converted from a text file (output of the recording program) into a MATLAB file. Next, the data were bandpass filtered between 10 and 30 Hz to reduce artefacts such as baseline drift and electrical interferences to improve the identification of R-peaks in the ECG. The filtered data were used as input to the MATLAB toolbox “HRVTool” [33] for calculation of HRV parameters. The HRVTool automatically identified the R-peaks, but all recordings were visually inspected to make sure that artefacts were not registered as R-peaks. If artefacts were present, they were corrected manually since artefacts would lead to incorrect R-R intervals and hence incorrect HRV-parameters. The extracted HRV measures included in the further analysis were the time domain parameters: (a) The standard deviation of the R-R intervals (SDNN), (b) the root mean square of successive R-R intervals (RMSSD), and (c) the proportion of NN50 (pairs of successive R-R intervals that differ more than 50 milliseconds) divided by the total number of R-R intervals (PNN50). In short-term ECG-recordings (5 min) all three parameters are parasympathetically-mediated [9]. The analysis was performed in MATLAB R2021a (MathWorks^®^, Natick, MA, USA).

### 2.5. Statistical Analysis

A power calculation was conducted based on data from a previous study [22]. Using within factors repeated measures Analysis of Variance (ANOVA), power 0.8, alpha 0.05, and effect size 0.546, the required sample size was 42 participants in each group. Data were tested for normal distribution, and if the assumption of normality was violated, a logarithm with base 10 was applied for SDNN and RMSSD, while a square root was applied to PNN50 since several measurements were zero. A Paired T-test or a Wilcoxon Signed Rank Test was used to investigate if there was a difference between pre1 and pre2 (baseline reliability), and if there was no significant change between the two, a common baseline was calculated as the mean of pre1 and pre2. A One-Way repeated measures ANOVA test with time as factor (4 levels: Baseline, post1, post2, post3) was used to investigate the effect of DB. This test was performed for each of the three HRV-parameters, and in healthy participants repeated for the three doses (5, 15, and 30 min) and in patients with RA and SLE repeated on intervention day 1 and day 2, i.e., 3 × 3 and 3 × 2 repeated measures ANOVA tests, respectively. If the assumption of sphericity was violated, a Greenhouse–Geisser correction was applied. Finally, a least significant difference post hoc test was used to identify significant changes in the pairwise comparisons. In both groups a percentage change was calculated for each HRV-parameter between baseline and post1, post2, post3, respectively, and they were used to: (1) analyse if 5, 15, or 30 min were the optimal dose of DB in healthy participants (using a One-Way repeated measures ANOVA with time as factor (3 levels: Baseline-post ${}_{5min}$, baseline-post${}_{15min}$, baseline-post${}_{30min}$)), and (2) to investigate the reliability between day 1 and day 2 in patients with RA and SLE (using a Paired T-test or a Wilcoxon Signed Rank Test). A *p*-value < 0.05 was considered statistically significant. All statistical analyses were performed in SPSS Statistics version 27.0 (IBM Corp., Armonk, NY, USA).

## 3. Results

### 3.1. Baseline Reliability

No significant change was found between pre1 and pre2 measurements in SDNN, RMSSD, or PNN50 for the healthy participants and patients, indicating that the HRV-parameters did not change while resting (*p*-values ranged from 0.140 to 0.901). Afterwards a mean baseline was used.

### 3.2. Results in Healthy Participants

#### 3.2.1. Effect of Deep Breathing

**Figure 1:**
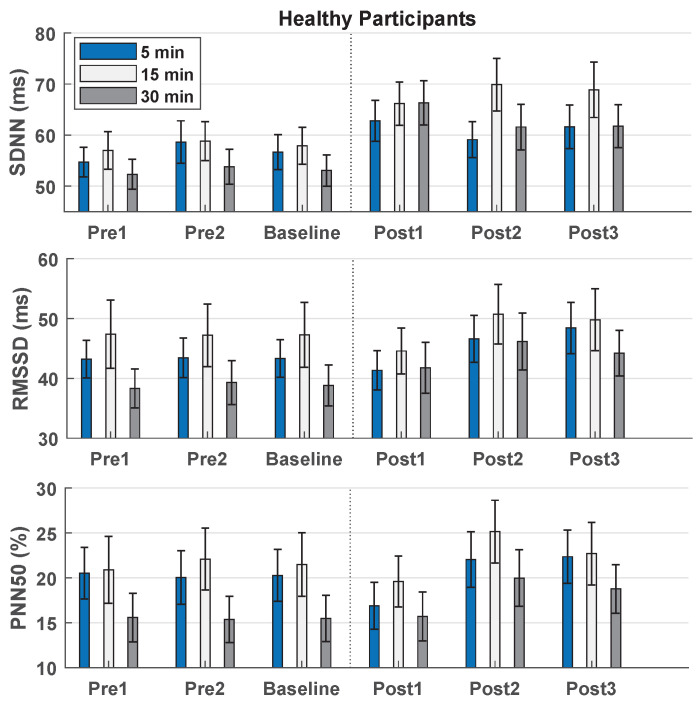
Bar chart showing the heart rate variability-parameters plotted as mean ± standard error in healthy participants. SDNN: The standard deviation of the R-R intervals. RMSSD: The root mean square of successive R-R intervals. PNN50: The proportion of NN50 (pairs of successive R-R intervals that differ more than 50 milliseconds) divided by the total number of R-R intervals. Post1, post2, and post3 were performed at 0–5, 12.5–17.5, and 25–30 min after the deep breathing intervention, respectively.

The effect of the intervention on HRV-parameters from baseline to post-measurements is summarized in Figure 1, Table 3 and Table 4. For all doses of DB, a significant effect of time was observed. For 5 min of DB only one significant increase was found between baseline and post measurements, regarding SDNN and RMSSD. For 15 min of DB, SDNN was significantly higher at all post measurements compared to baseline, while RMSSD and PNN50 were significantly higher at one of the post measurements each. For 30 min of DB, SDNN was significantly higher at all post measurements compared to baseline, while RMSSD and PNN50 were significantly higher at two and one of the post measurements, respectively. The effect of the intervention remained or increased throughout the post-measurements indicating that the effects outlasted the intervention period, regarding both SDNN, RMSSD, and PNN50. Only two significant decreases were found, see Table 3.

#### 3.2.2. Interventional Dose

When comparing 5, 15, and 30 min of DB, there was a significant change in SDNN in the percentage change between baseline and post1 (F(1.675, 65.334) = 3.622, *p* = 0.040 *). The post hoc test revealed a significant change between 5 and 30 min (*p* = 0.037 *) and between 15 and 30 min (*p* = 0.047 *), with 30 min being associated with higher increases in HRV. Another change was found in SDNN between baseline and post2 (F(1.590, 61.993) = 3.020, *p* = 0.067), where the post hoc test showed one significant change between 5 and 15 min (*p* = 0.002 *). No significant changes were found in RMSSD or PNN50. The percentage change between baseline and post-measurements for SDNN ranged from 8 to 13% after 5 min of DB, 17 to 23% after 15 min of DB, and 18 to 32% after 30 min of DB. Thus, the greatest percentage increase in HRV-parameters was after 30 min of DB and therefore, 30 min of DB was chosen as the interventional dose for investigating across-days reliability in patients with RA and SLE.

### 3.3. Results in Patients with RA and SLE

#### 3.3.1. Effect of Deep Breathing

**Figure 2:**
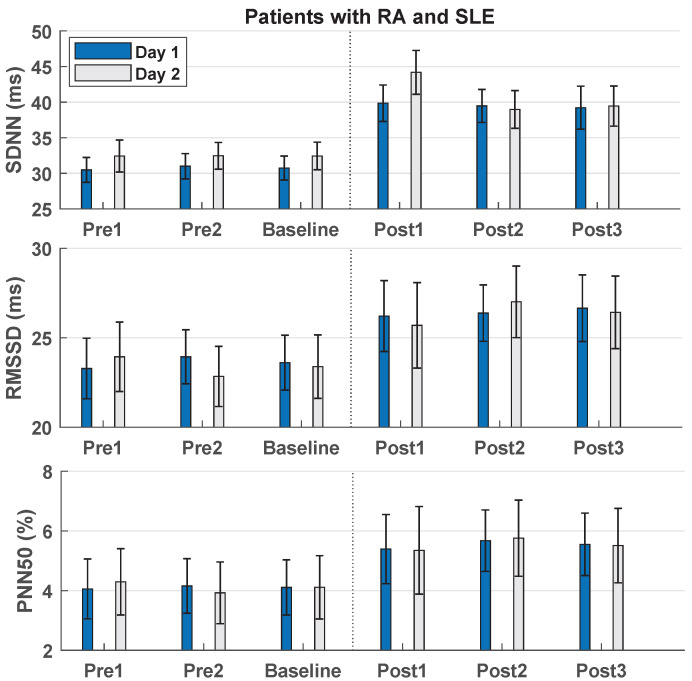
Bar chart showing the heart rate variability-parameters plotted as mean ± standard error in patients with RA and SLE. RA: Rheumatoid Arthritis. SLE: Systemic Lupus Erythematosus. SDNN: The standard deviation of the R-R intervals. RMSSD: The root mean square of successive R-R intervals. PNN50: The proportion of NN50 (pairs of successive R-R intervals that differ more than 50 milliseconds) divided by the total number of R-R intervals. Post1, post2, and post3 were performed at 0–5, 12.5–17.5, and 25–30 min after the deep breathing intervention, respectively.

The effect of 30 min of DB on HRV-parameters from baseline to post-measurements is summarized in Figure 2, Table 4 and Table 5. For day 1 there was a significant increase in the three HRV-parameters in all post-measurements, except one pairwise comparison regarding PNN50. For day 2 there was a significant increase in the three HRV-parameters in all post-measurements, except two pairwise comparisons regarding RMSSD and PNN50. The effect of the intervention remained throughout the post-measurements, since only two significant decreases were found, see Table 5, indicating that the effect outlasted the intervention period.

The effect of deep breathing on HRV in healthy participants reported with a one-way repeated measures ANOVA and *p*-values of the post hoc test. A significant effect of time was seen for all three doses (5, 15, and 30 min). SDNN, RMSSD, and PNN50 increased from baseline to some post measurements with a dose of 5 or 15 min while 30 min of deep breathing increased SDNN, RMSSD, and PNN50 from baseline to most post measurements.

#### 3.3.2. Test-Retest Reliability

No significant change was found between intervention day 1 and intervention day 2 (*p*-values ranged from 0.290 to 0.935), indicating reliability of the intervention from baseline to post-measurements across days.

## 4. Discussion

In this study the optimal dose of DB in healthy participants and the reliability of the effect of DB on HRV in patients with RA and SLE were investigated for the first time. No significant change was found in the measurements obtained before the intervention, indicating that HRV-parameters do not change while resting. An increase in HRV-parameters was found in healthy participants already after 15 min of DB, but more certainly after 30 min of DB. In patients with RA and SLE, 30 min of DB increased all HRV-parameters, however, the effect on PNN50 had a slower onset. The findings indicated reliability between the two intervention days, and the effect remained throughout the post-measurements, indicating that the effects outlasted the VNS period.

Looking at the baseline measurements, the patients with RA and SLE had lower SDNN (31 milliseconds) and RMSSD (24 milliseconds) compared to normal values reported for healthy adults (SDNN: 50 milliseconds and RMSSD: 42 milliseconds) [34]. The healthy participants in this study had a baseline SDNN (57 milliseconds) and RMSSD (43 milliseconds) comparable to normal values in healthy adults [34]. This supports the fact that patients with RA and SLE have a lower tone of the autonomic nervous system measured through HRV, and other studies confirm similar results of depressed HRV [7,8,15].

Thirty minutes of DB increased HRV-parameters in both healthy participants and in patients with RA and SLE. Previous studies in healthy participants (see Table 6 for an overview) also found an increase in HRV after yoga and breathing exercises [24,25,26,35]. However, a study in healthy participants by Sasaki et al. found that an HRV-parameter of parasympathetic nerve activity decreased during controlled breathing [27]. It is difficult to compare results across the different studies since they had different overall aims. In addition, different measures of vagal tone have been used as well as differences in breathing parameters such as inspiration–expiration ratio and duration of the DB intervention, but generally DB interventions have been associated with increased vagal tone and parasympathetic activity. Moreover, other factors can influence HRV such as (1) the length of the recording period (longer recordings are associated with increased HRV), (2) removal of artefacts (e.g., extrasystoles) and (3) age, gender, heart rate, health status, and comorbidities [9,36]. In patients with RA and SLE, the findings in the current study are in agreement with a similar study using the same intervention and HRV-parameters [22]. Similar increases in SDNN and PNN50 were observed, but the absolute RMSSD values were slightly lower in the current study, which could be attributed to inter-individual differences.

To our knowledge, no other studies examined the dose-response relationship of DB on HRV in healthy participants. One study found an increase in HRV during breathing at a frequency of 5–7 breaths/minute for 2 min [10] and another study during 10 min of guided breathing exercises [25]. However, both studies measured HRV during the breathing exercises, which is debatable since respiration itself can be a confounding factor in HRV evaluation [37]. The influence of respiration on HRV is a phenomenon known as respiratory sinus arrhythmia (RSA), and it is characterized by shortening of the RR-interval during inspiration and prolongation of the RR-interval during expiration [38]. In this work HRV was measured after the intervention to avoid the direct influence of RSA.

tVNS is another possible way of stimulating the vagus nerve non-invasively. A study in healthy participants found that 15 min of auricular tVNS increased HRV [39]. Brock et al. investigated cervical tVNS for 120 s, 3 times a day, for 5 consecutive days in patients with psoriatic arthritis and found a reduction in disease activity and a 20% reduction of CRP [40]. A pilot study of tVNS in patients with RA showed similar results [41]. These studies of tVNS could indicate that the required amount of stimulation time is smaller when using tVNS compared to DB. Compliance may also be better ensured when using tVNS, since 30 min of DB demands complete concentration.

The invasive alternative to DB and tVNS is an implantable vagus nerve stimulator, which was investigated in a study by Koopman et al. [6]. They found that an implantable vagus nerve stimulator could inhibit tumour necrosis factor and interleukin-6 production and improve disease severity in patients with RA, indicating a possible correlation between the immune system and the autonomic nervous system [6]. However, an implantable vagus nerve stimulator has known side effects such as hoarseness, dysphonia, and coughing [6], so physiological methods such as DB have some clear advantages such as accessibility, fewer side effects, and no costs.

The present study found increased HRV-parameters after DB in patients with RA and SLE, indicating a higher parasympathetic tone, which may have implications for the management and treatment of RA and SLE. It has been reported that autonomic imbalance precedes the development of RA in individuals at risk of developing arthritis [11], and patients with more vagal activity respond better to anti-tumour necrosis factor treatment [13]. Thus, DB could potentially be used to make RA and SLE patients respond better to their medication and hence improve the management of the diseases or potentially prevent or postpone development of the diseases. However, these are just speculations and should be tested in future studies. It is not known if the vagus nerve and its anti-inflammatory properties are activated with resulting decreased levels of pro-inflammatory cytokines, e.g., tumour necrosis factor-α.

### Limitations and Future Perspectives

Five, 15, and 30 min of DB were investigated in healthy participants, while only 30 min of DB was investigated in patients. Consequently, it is unknown whether doses in between are effective, and future studies could investigate the dose-response more thoroughly both in patients and healthy participants by changing the interventional dose, e.g., 20 min and 25 min. In continuation of this, it is also important to consider how often and how long the DB should be performed, both concerning compliance and to maintain an adequate effect. It is still uncertain if the effect is sustained beyond 30 min, which is why further examination of the washout effect should be carried out. That could be done simply by adding additional measurements after 30 min.

This research area is still relatively unexplored, and taking the above-mentioned into consideration, future studies could be designed as longitudinal interventional studies, preferably as a randomized controlled trial with the control group performing un-paced (sham) breathing. The interventional effect could be examined in relation to clinical parameters such as disease activity scores and biochemical measures such as CRP and cytokine levels.

HRV is an indicator of cardiac vagal tone, which does not necessarily reflect the anti-inflammatory properties of the vagus nerve. It is not known whether the vagus nerve might be organ specific, meaning that vagal influence on the heart may not represent vagal input to inflammatory organs such as the spleen, lungs, gut, or liver as well [42]. Therefore, clinical, and biochemical measures could contribute to clarify the potential anti-inflammatory effect of DB. Moreover, future studies should include patients with remission, low-grade disease activity, and high-grade disease activity to make conclusions applicable for a wider spectrum of the patient groups. In addition, the intervention should be tested on a larger number of SLE patients to determine the efficacy of the intervention in this patient group alone since the majority of patients in the current study were diagnosed with RA, which was due to prevalence of RA and SLE patients.

In summary, the future research direction could be: (1) to investigate the parameters of the intervention to maximize the effect, (2) investigate the effect on disease activity and biochemical measures in randomized clinical trials, (3) investigate the exact physiological mechanisms associated with the DB intervention, and (4) investigate how adherence to the DB training can be maximized and made motivating and engaging to perform for the patients.

## 5. Conclusions

This dose-response study in healthy participants found that 30 min of DB was the optimal dose to increase HRV. In patients with RA and SLE, the study of reliability showed that 30 min of DB increased HRV, and the results indicated that the effect of DB was reliable across days. The findings suggest that the vagus nerve can be stimulated through DB, however the results need to be investigated in future studies to examine the effect of DB on inflammatory markers as well.

## Figures and Tables

**Table 1:** Inclusion and exclusion criteria. * Rheumatoid Arthritis according to the American College of Rheumatology (1987 or 2010) or European League Against Rheumatism (2010) classification criteria [28]. ** Systemic Lupus Erythematosus according to American College of Rheumatology classification criteria for SLE [29] or Systemic Lupus International Collaborating Clinics Classification [30].

Inclusion Criteria	Exclusion Criteria
Men and women in the age range from 18 years to 85 years	Heart arrhythmias
Healthy participants or	Chronic lung diseases
Rheumatoid Arthritis * or	Pregnancy
Systemic Lupus Erythematosus **	Severe mental illness
	Present or former addiction
	Being unable to provide informed consent

**Table 2:** Participant characteristics. RA: Rheumatoid Arthritis. SLE: Systemic Lupus Erythematosus. n: Number. SD: Standard deviation. BMI: Body mass index. DAS28-CRP: Disease Activity Score-28 CRP. CDAI: Clinical Disease Activity Index. SLE-DAI. Systemic Lupus Erythematosus Disease Activity Index. MDHAQ: The multidimensional health assessment questionnaire. SLAQ: Systemic Lupus Activity Questionnaire. cDMARD: Conventional DMARD. bDMARD: Biological DMARD. Anti-CCP: Anti-citrullinated protein antibody. ANA: Antinuclear antibody. Anti-dsDNA: Anti-double stranded DNA. CRP: C-reactive protein.

Characteristics	Healthy	RA	SLE
Participants, n.	40	45	6
Sex, n. (female)	22	32	4
Sex, n. (male)	18	13	2
Age, mean ± SD (years)	32 ± 13	58 ± 13	53 ± 21
BMI, mean ± SD (kg/m^2^)	23 ± 3	26 ± 4	25 ± 6
Time of diagnosis, mean ± SD (year)	-	2009 ± 10	2009 ± 16

Disease activity scores, mean ± SD			
DAS28-CRP	-	2.39 ± 1.11	-
CDAI	-	6.03 ± 7.33	-
SLE-DAI	-	-	6.33 ± 4.27

Patient-reported outcome measures			
MDHAQ	-	0.39 ± 0.35	0.20 ± 0.39
SLAQ	-	-	8.86 ± 10.47

Treatment, n. receiving			
cDMARD	-	35	4
bDMARD	-	26	0
Prednisolone	-	0	3

Biomarkers, n. positive			
Rheuma factor	-	33	-
Anti-CCP	-	30	-
ANA	-	-	2
Anti-dsDNA	-	-	3

CRP, mean ± SD (mg/L)	-	5.11 ± 8.84	4.48 ± 2.82

**Table 3:** The effect of deep breathing on HRV in healthy participants reported with a one-way repeated measures ANOVA and *p*-values of the post hoc test. A significant effect of time was seen for all three doses (5, 15, and 30 min). SDNN, RMSSD, and PNN50 increased from baseline to some post measurements with a dose of 5 or 15 min while 30 min of deep breathing increased SDNN, RMSSD, and PNN50 from baseline to most post measurements. HRV: Heart rate variability. ANOVA: Analysis of Variance. * Shows statistical significance. ↑ Shows an increase with respect to the baseline. ↓ Shows a decrease with respect to the baseline. The left column shows the name of the HRV-measure and each of the three interventional doses that were tested: 5, 15, and 30 min. Statistics: F(df (Time), df (Error (Time))) = F-value, *p*-value. SDNN: The standard deviation of the R-R intervals. RMSSD: The root mean square of successive R-R intervals. PNN50: The proportion of NN50 (pairs of successive R-R intervals that differ more than 50 milliseconds) divided by the total number of R-R intervals. Post1, post2, and post3 were performed at 0–5, 12.5–17.5, and 25–30 min after the deep breathing intervention, respectively. “B_PX” refers to the comparison between baseline and post1, post2, or post3. “PX_PX” refers to the comparison between post1, post2, or post3.

	Statistics	B_P1	B_P2	B_P3	P1_P2	P1_P3	P2_P3
Log10(SDNN)							
5 min.	F(3, 117) = 2.786, *p* = 0.044 *	0.006 * ↑	0.169 ↑	0.054 ↑	0.167 ↓	0.436 ↓	0.536 ↑
15 min.	F(2.557, 99.725) = 8.233, *p* < 0.001 *	0.001 * ↑	<0.001 * ↑	0.001 * ↑	0.260 ↑	0.738 ↑	0.316 ↓
30 min.	F(3, 117) = 6.326, *p* = 0.001 *	<0.001 * ↑	0.031 * ↑	0.010 * ↑	0.037 * ↓	0.135 ↓	0.648 ↑
Log10(RMSSD)							
5 min.	F(2.482, 96.790) = 6.032, *p* = 0.002 *	0.119 ↓	0.075 ↑	0.026 * ↑	0.007 * ↑	0.002 * ↑	0.317 ↑
15 min.	F(2.587, 100.905) = 3.529, *p* = 0.023 *	0.578 ↓	0.038 * ↑	0.103 ↑	0.012 * ↑	0.056 ↑	0.258 ↓
30 min.	F(2.542, 99.122) = 3.087, *p* = 0.038 *	0.454 ↑	0.030 * ↑	0.020 * ↑	0.058 ↑	0.142 ↑	0.981 ↓
$\sqrt{\left(PNN50\right)}$							
5 min.	F(3, 117) = 6.246, *p* = 0.001 *	0.022 * ↓	0.185 ↑	0.187 ↑	0.002 * ↑	0.002 * ↑	0.753 ↑
15 min.	F(3, 117) = 4.488, *p* = 0.005 *	0.335 ↓	0.024 * ↑	0.540 ↑	0.002 * ↑	0.128 ↑	0.017 * ↓
30 min.	F(2.553, 99.576) = 3.364, *p* = 0.028 *	0.958 ↓	0.036 * ↑	0.062 ↑	0.003 * ↑	0.066 ↑	0.704 ↓

**Table 4:** Heart rate variability-parameters reported as mean ± standard error across subjects in the dose-response sub-study with healthy subjects and in the test-retest reliability study with RA and SLE patients. RA: Rheumatoid Arthritis. SLE: Systemic Lupus Erythematosus. SDNN: The standard deviation of the R-R intervals. RMSSD: The root mean square of successive R-R intervals. PNN50: The proportion of NN50 (pairs of successive R-R intervals that differ more than 50 milliseconds) divided by the total number of R-R intervals. Post1, post2, and post3 were performed at 0–5, 12.5–17.5, and 25–30 min after the deep breathing intervention, respectively.

	SDNN (ms)	RMSSD (ms)	PNN50 (%)
Healthy	[5 | 15 | 30 min]	[5 | 15 | 30 min]	[5 | 15 | 30 min]
Pre1	54.7 ± 2.9 | 57.0 ± 3.7 | 52.3 ± 3.0	43.2 ± 3.1 | 47.4 ± 5.7 | 38.3 ± 3.2	20.5 ± 2.9 | 20.9 ± 3.7 | 15.6 ± 2.7
Pre2	58.6 ± 4.2 | 58.8 ± 3.8 | 53.8 ± 3.4	43.4 ± 3.3 | 47.2 ± 5.2 | 39.3 ± 3.7	20.0 ± 3.0 | 22.1 ± 3.4 | 15.4 ± 2.6
Baseline	56.7 ± 3.4 | 57.9 ± 3.6 | 53.1 ± 3.1	43.3 ± 3.2 | 47.3 ± 5.4 | 38.8 ± 3.4	20.3 ± 2.9 | 21.5 ± 3.5 | 15.5 ± 2.6
Post1	62.8 ± 4.0 | 66.2 ± 4.2 | 66.3 ± 4.4	41.3 ± 3.3 | 44.6 ± 3.8 | 41.8 ± 4.3	16.9 ± 2.6 | 19.6 ± 2.8 | 15.7 ± 2.7
Post2	59.1 ± 3.5 | 69.9 ± 5.2 | 61.6 ± 4.5	46.6 ± 3.9 | 50.7 ± 5.0 | 46.1 ± 4.8	22.1 ± 3.1 | 25.1 ± 3.5 | 22.7 ± 3.5
Post3	61.6 ± 4.3 | 68.9 ± 5.4 | 61.7 ± 4.2	48.4 ± 4.3 | 49.8 ± 5.2 | 44.2 ± 3.8	22.4 ± 3.0 | 22.7 ± 3.5 | 18.8 ± 2.7

RA and SLE	[Day 1 | Day 2]	[Day 1 | Day 2]	[Day 1 | Day 2]
Pre1	30.5 ± 1.7 | 32.4 ± 2.2	23.3 ± 1.7 | 23.9 ± 1.9	4.1 ± 1.0 | 4.3 ± 1.1
Pre2	31.0 ± 1.8 | 32.5 ± 1.9	23.9 ± 1.5 | 22.8 ± 1.7	4.2 ± 0.9 | 3.9 ± 1.0
Baseline	30.7 ± 1.7 | 32.4 ± 1.9	23.6 ± 1.5 | 23.4 ± 1.8	4.1 ± 0.9 | 4.1 ± 1.1
Post1	39.8 ± 2.6 | 44.2 ± 3.1	26.2 ± 2.0 | 25.7 ± 2.4	5.4 ± 1.2 | 5.3 ± 1.5
Post2	39.5 ± 2.3 | 39.0 ± 2.6	26.4 ± 1.6 | 27.0 ± 2.0	5.7 ± 1.0 | 5.8 ± 1.3
Post3	39.2 ± 3.0 | 39.5 ± 2.8	26.7 ± 1.9 | 26.4 ± 2.0	5.6 ± 1.0 | 5.5 ± 1.2

**Table 5:** The effect of deep breathing on HRV in patients with RA and SLE reported with a one-way repeated measures ANOVA and *p*-values of the post hoc test. A significant increase was observed from baseline to post measurements for SDNN, RMSSD, and PNN50 on both days, and the HRV-parameters remained elevated throughout the post measurements. HRV: Heart rate variability. RA: Rheumatoid Arthritis. SLE: Systemic Lupus Erythematosus. ANOVA: Analysis of Variance. * Shows statistical significance. ↑ Shows an increase with respect to the baseline. ↓ Shows a decrease with respect to the baseline. Statistics: F(df (Time), df (Error (Time))) = F-value, *p*-value. SDNN: The standard deviation of the R-R intervals. RMSSD: The root mean square of successive R-R intervals. PNN50: The proportion of NN50 (pairs of successive R-R intervals that differ more than 50 milliseconds) divided by the total number of R-R intervals. Post1, post2, and post3 were performed at 0–5, 12.5–17.5, and 25–30 min after the deep breathing intervention, respectively. “B_PX” refers to the comparison between baseline and post1, post2, or post3. “PX_PX” refers to the comparison between post1, post2, or post3.

	Statistics	B_P1	B_P2	B_P3	P1_P2	P1_P3	P2_P3
Log10(SDNN)							
Day 1	F(3, 150) = 11.455, *p* < 0.001 *	<0.001 * ↑	<0.001 * ↑	<0.001 * ↑	0.995 ↓	0.556 ↓	0.540 ↓
Day 2	F(2.367, 118.358) = 13.120, *p* < 0.001 *	<0.001 * ↑	<0.001 * ↑	<0.001 * ↑	0.027 * ↓	0.029 * ↓	0.787 ↑
Log10(RMSSD)							
Day 1	F(2.586, 129.302) = 4.470, *p* = 0.008 *	0.024 * ↑	0.004 * ↑	<0.001 * ↑	0.523 ↑	0.490 ↑	0.989 ↓
Day 2	F(2.611, 130.540) = 5.143, *p* = 0.003 *	0.094 ↑	0.001 * ↑	0.003 * ↑	0.080 ↑	0.273 ↑	0.425 ↓
$\sqrt{\left(PNN50\right)}$							
Day 1	F(2.670, 133.485) = 3.738, *p* = 0.016 *	0.074 ↑	0.009 * ↑	0.003 * ↑	0.246 ↑	0.463 ↑	0.672 ↓
Day 2	F(2.078, 103.904) = 3.385, *p* = 0.036 *	0.060 ↑	0.007 * ↑	0.014 * ↑	0.300 ↑	0.518 ↑	0.495 ↓

**Table 6:** Overview of representative deep/controlled breathing studies.

Study	Subjects	Intervention	HRV Measures Main Findings	
Current study	RA (n = 45), SLE (n = 6)	DB: 30 min	SDNN, RMSSD, PNN50	Increase in all parameters after DB with respect to a baseline recorded prior DB on two separate days.
Rovsing et al. [22]	RA (n = 49), SLE (n = 8)	DB: 30 min	SDNN, RMSSD, PNN50	Increase in all parameters after DB with respect to a baseline recorded prior DB.
Muralikrishnan et al. [26]	Yoga (n = 14), Non-yoga (n = 14)	DB: 1 min	SDNN, RMSSD, PNN50, LF, and HF power	No difference between the two groups at baseline, but after DB SDNN, RMSSD, HF, and PNN50 increased while LF decreased in the Yoga-practitioner group.
Sharpe et al. [24]	Healthy (n = 25)	DB (self-paced): 2 × 10 min DB (externally-paced): 2 × 10 min	SDNN, RMSSD, LF, and HF power	Increase in SDNN and RMSSD after externally-paced DB with respect to a baseline (regular breathing).
Tanriverdi et al. [35]	Healthy (n = 36)	Diaphragmatic breathing: 15 min	SNN, RMSSD, PNN50, LF, and HF power	Increase in SDNN, RMSSD, and PNN50 after diaphragmatic breathing with respect to a baseline recorded prior diaphragmatic breathing.
Sasaki et al. [27]	Healthy (n = 20)	Controlled breathing: 5 min	LF and HF power	Decrease in HF power after controlled breathing compared to regular breathing.

## Data Availability

The data presented in this study are available on request from the corresponding author.
